# Supplementary material for: Nucleotide context models outperform protein language models for predicting antibody affinity maturation
Source: PLoS Comput Biol. 2025 Dec 1;21(12):e1013758. doi: 10.1371/journal.pcbi.1013758 (PMC12685221; doi:10.1371/journal.pcbi.1013758)
Supplement: S1 Text — File containing all supplementary tables and figures. (PDF) [file pcbi.1013758.s001.pdf]

Supplementary Materials

**Data set**	**Species**	**PCPs**	**Clonal families**	**Median PCPs/CF**	**Median subs./PCP**	**Comments**
Tang et al. IgH [11]	Human	438,302	130,621	2	2	In OAS (AbLang training set)
Jaffe et al. IgH [7]	Human	176,656	49,430	2	4	In OAS (AbLang training set) and Thrifty-prod training set
Rodriguez et al. IgH [6]	Human	18,639	6,837	2	4	Not in OAS or any training set
Ford et al. IgH [5]	Human	2,407	1,182	2	3	Not in OAS or any training set
Replay IgH [8]	Mouse	5,378	129	44	1	Not in OAS or any training set
Replay Igκ [8]	Mouse	5,176	129	40	1	Not in OAS or any training set

Table A. Summary of data sets used. All human data results in the main text are based on the Tang et al. IgH data set.

Model	Overlap	R-prec.	Sub. Acc.	CSP Perp.
S5F	0.782	0.118	0.263	8.20
S5F + ESM-1v	0.617	0.112	0.328	9.12
S5F + BLOSUM62	0.795	0.122	0.300	7.84
Thrifty-SHM	0.823	0.145	0.326	6.87
Thrifty-SHM + ESM-1v	0.624	0.116	0.334	8.63
Thrifty-SHM + BLOSUM62	0.835	0.149	0.350	6.67
Thrifty-prod	**0.900**	**0.163**	**0.370**	**6.36**
ESM-1v	0.739	0.091	0.252	13.48
AbLang2	0.780	0.095	0.353	8.20

Table B. Model performance on Rodriguez et al. IgH data set. The best model for each metric is indicated in **bold**. The Rodriguez et al. data set is not part of any model training data.

Model	Overlap	R-prec.	Sub. Acc.	CSP Perp.
S5F	0.779	0.106	0.261	8.33
S5F + ESM-1v	0.613	0.096	0.324	9.61
S5F + BLOSUM62	0.790	0.107	0.300	8.01
Thrifty-SHM	0.824	0.128	0.324	7.00
Thrifty-SHM + ESM-1v	0.620	0.102	0.328	8.99
Thrifty-SHM + BLOSUM62	0.834	0.132	0.345	6.80
Thrifty-prod	**0.900**	**0.141**	**0.365**	**6.52**
ESM-1v	0.733	0.082	0.249	13.72
AbLang2	0.787	0.090	0.332	8.61

Table C. Model performance on Ford et al. IgH data set. The best model for each metric is indicated in **bold**. The Ford et al. data set is not part of any model training data.

Model	Overlap	R-prec.	Sub. Acc.	CSP Perp.
S5F	0.783	0.096	0.262	8.19
S5F + ESM-1v	0.616	0.100	0.326	9.11
S5F + BLOSUM62	0.786	0.098	0.299	7.92
Thrifty-SHM	0.834	0.127	0.328	6.86
Thrifty-SHM + ESM-1v	0.627	0.103	0.331	8.65
Thrifty-SHM + BLOSUM62	0.836	0.129	0.344	6.73
Thrifty-prod	**0.923**	**0.139**	**0.363**	**6.46**
ESM-1v	0.744	0.082	0.255	12.61
AbLang2	0.774	0.097	0.356	7.61

Table D. Model performance on the Tang et al. IgH data set. The best model for each metric is indicated in **bold**. The Tang et al. data set is part of the AbLang2 training data.

Model	Overlap	R-prec.	Sub. Acc.	CSP Perp.
S5F	0.771	0.111	0.277	7.38
S5F + ESM-1v	0.595	0.099	0.341	8.10
S5F + BLOSUM62	0.780	0.113	0.319	7.01
Thrifty-SHM	0.818	0.147	0.347	6.22
Thrifty-SHM + ESM-1v	0.603	0.107	0.345	7.73
Thrifty-SHM + BLOSUM62	0.823	0.150	0.369	6.00
Thrifty-prod	**0.907**	**0.164**	0.393	**5.66**
ESM-1v	0.710	0.073	0.258	12.43
AbLang2	0.754	0.086	**0.41**	6.23

Table E. Model performance on Jaffe et al. IgH data set. The best model for each metric is indicated in **bold**. The Jaffe et al. data set is the training data for Thrifty-prod and part of the training data for AbLang2.

**Model**	**Modeling perspective**	**Biological process**	**EPAM framework**	**Species evaluated in EPAM**
Thrifty-SHM [2]	ME	SHM	NT	Human
Thrifty-prod [2]	ME	Implicit SHM-selection	NT	Human
S5F [1]	ME	SHM	NT	Human
S5F + ESM-1v	ME+LM	Explicit SHM-selection	NT-AA	Human
S5F + BLOSUM62 [12]	ME	Explicit SHM-selection	NT-AA	Human
Thrifty-SHM + ESM-1v	ME+LM	Explicit SHM-selection	NT-AA	Human
Thrifty-SHM + BLOSUM62	ME	Explicit SHM-selection	NT-AA	Human
ESM-1v (model 1) [9]	LM	Selection	AA	Human, mouse
AbLang [3,4]	LM	Implicit SHM-selection	AA	Human, mouse
ReplaySHM [8]	ME	SHM	NT	Mouse
ReplaySHM + DMS	ME	Explicit SHM-selection	NT-AA	Mouse
ReplaySHM + BLOSUM62	ME	Explicit SHM-selection	NT-AA	Mouse
ReplaySHM + ESM-1v	ME+LM	Explicit SHM-selection	NT-AA	Mouse

Table F. Classifications for all models considered. Models are characterized by the modeling perspective considered: molecular evolution (ME) or language modeling (LM). The molecular evolution perspective includes mutation models (SHM) and models that combine mutation and selection (explicit SHM-selection), while language models either characterize the joint substitution process (implicit SHM-selection) or selection alone. EPAM frameworks are classified by the input data type, considering nucleotide (NT) and/or amino acid (AA) sequences. Models are evaluated on human and mouse data sets in EPAM where applicable.

	23	41	43	98	102	104	118
Observed	0.14	0.15	0.13	0.19	0.25	0	0
S5F	0.77	0.46	0.33	0.60	0.98	0.72	0.43
Thrifty-SHM	0.54	0.51	0.39	0.49	0.85	0.61	0.43
ESM-1v	0.11	0.11	0.15	0.11	0.14	0.11	0.16
Thrifty-SHM + ESM-1v	0.09	0.09	0.10	0.10	0.11	0.09	0.10
AbLang2	0.12	0.27	0.25	0.30	0.46	0.12	0.39
Thrifty-prod	0.27	0.27	0.22	0.23	0.49	0.27	0.23

Table G. Quantile of the observed or expected number of substitutions at known conserved sites in the Tang et al. IgH data set.

DMS data	chain	Overlap	R-prec.	Sub. Acc.	CSP Perp.
bind	IgH	0.820	0.146	0.430	5.10
expr	IgH	0.797	0.143	0.420	5.06
bind+expr	IgH	0.821	0.146	0.430	5.09
bind	Igκ	0.786	0.125	0.423	4.77
expr	Igκ	0.725	0.090	0.386	4.88
bind+expr	Igκ	0.789	0.125	0.423	4.77

Table H. Incorporating expression measurements for ReplaySHM+DMS. Performance comparisons for different sources of DMS measurements to compute selection factors for the ReplaySHM + DMS model. For binding measurements (bind), as reported in the main results, selection factors are the ratio of dissociation contants, $R = K_D^{\text{parent}}/K_D^{\text{child}}$. For expression measurements (expr), the selection factors are the ratio of expression values, $R = E^{\text{child}}/E^{\text{parent}}$. For combining binding and expression (bind+expr), the selection factors are computed as, $R = (K_D^{\text{parent}}/K_D^{\text{child}})\,(E^{\text{child}}/E^{\text{parent}})$.

	IgH			Igκ		
	naive	leaf	all	naive	leaf	all
No. of PCPs	391	4096	5378	424	3772	5176
No. of amino acid substitutions	595	6772	8499	702	6094	8107
overlap: ReplaySHM	0.654	0.822	0.790	0.545	0.751	0.717
overlap: ReplaySHM + DMS	0.649	0.847	0.820	0.580	0.820	0.786
overlap: relative change (%)	-0.8	+3.0	+3.7	+6.6	+9.2	+9.6
R-prec: ReplaySHM	0.161	0.128	0.143	0.045	0.088	0.090
R-prec: ReplaySHM + DMS	0.158	0.131	0.146	0.103	0.118	0.125
R-prec: relative change (%)	-1.8	+2.4	+2.4	+126	+33	+38
sub. acc.: ReplaySHM	0.316	0.411	0.416	0.281	0.406	0.386
sub. acc.: ReplaySHM + DMS	0.365	0.422	0.430	0.400	0.423	0.423
sub. acc.: relative change (%)	+15	+2.7	+3.4	+43	+4.2	+9.5
CSP perp.: ReplaySHM	6.324	5.035	5.070	5.543	4.859	4.907
CSP perp.: ReplaySHM + DMS	5.116	5.269	5.096	4.673	4.951	4.772
CSP perp.: change	-1.207	+0.234	+0.026	-0.870	+0.092	-0.135

Table I. Performance comparisons of ReplaySHM versus ReplaySHM + DMS on naive-parent PCPs and leaf-child PCPs. The data set is split into PCPs with parent sequence being the naive sequence (the "naive" columns) and PCPs with child sequence being a leaf node (the "leaf" columns). Most of the data are in leaf-child PCPs. Performance metrics for the ReplaySHM model and the ReplaySHM + DMS model are reported, with change in performance compared to ReplaySHM. The "relative change" is the difference in the metric divided by the ReplaySHM value, reported as a percentage. For CSP perplexity, the absolute difference is reported; a lower CSP perplexity score is better and a negative difference indicates an improvement.

**Model**	**Overlap**	**R-prec.**	**Sub. Acc.**	**CSP Perp.**
ReplaySHM	0.790	0.143	0.416	5.07
ReplaySHM + BLOSUM62	0.777	0.129	0.420	**5.01**
ReplaySHM + DMS	**0.820**	**0.146**	**0.430**	5.10
ReplaySHM + ESM-1v	0.535	0.034	0.374	7.69
ESM-1v	0.530	0.011	0.212	15.9
AbLang2	0.623	0.023	0.385	5.88

Table J. Model performance on Replay IgH data set. The best model for each metric is indicated in **bold**.

**Model**	**Overlap**	**R-prec.**	**Sub. Acc.**	**CSP Perp.**
ReplaySHM	0.717	0.090	0.386	4.91
ReplaySHM + BLOSUM62	0.710	0.091	0.419	4.79
ReplaySHM + DMS	**0.786**	**0.125**	**0.423**	**4.77**
ReplaySHM + ESM-1v	0.550	0.039	0.377	8.70
ESM-1v	0.547	0.017	0.182	21.24
AbLang2	0.646	0.022	0.367	7.30

Table K. Model performance on Replay Igκ data set. The best model for each metric is indicated in **bold**.

**Model**	**IgH aff.**	**Igκ aff.**	**IgH expr.**	**Igκ expr.**
ESM-1v 1	0.0317	0.2636	0.3701	0.5538
ESM-1v 2	0.0466	0.2677	0.4016	0.5739
ESM-1v 3	0.0438	0.2414	0.3890	0.5284
ESM-1v 4	0.0527	0.2427	0.4163	0.5688
ESM-1v 5	0.0421	0.2551	0.3788	0.5699
ESM-1v ensemble	0.0477	0.2658	0.4076	0.5793

Table L. DMS and ESM-1v correlations for Replay IgH and Igκ naive sequences. The ESM-1v values used here are the log transformed probability ratios (site-specific probabilities relative to the wildtype amino acid). Correlation values represent the Spearman rank correlation coefficient between DMS affinity or expression measures and ESM-1v predictions. We observed a stronger correlation between ESM-1v and DMS expression values relative to affinity. Similar trends were found in large benchmarks of DMS measurements and ESM models [10].

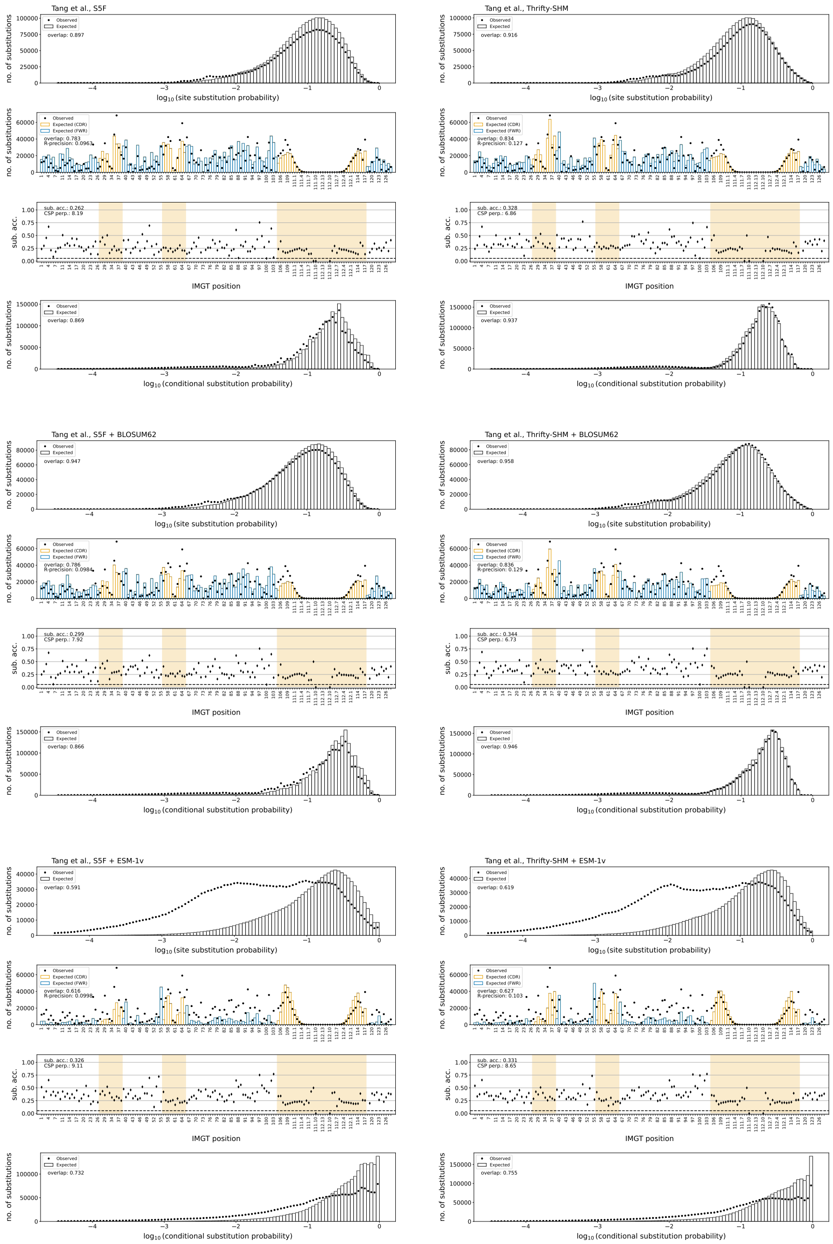

Fig A. NT and NT-AA framework models on the Tang et al. data set. For each model, there are four panels. The top panel shows the observed and expected number of substitutions where the sites are binned according to their predicted SSP. The second panel shows the observed and expected number of substitutions over site position. The third panel shows the per-site substitution accuracy. The bottom panel shows the observed and expected number of substitutions at sites of substitution, and the sites are binned according to their predicted CSP. The hatch-filled bands are the standard deviations on the expectations.

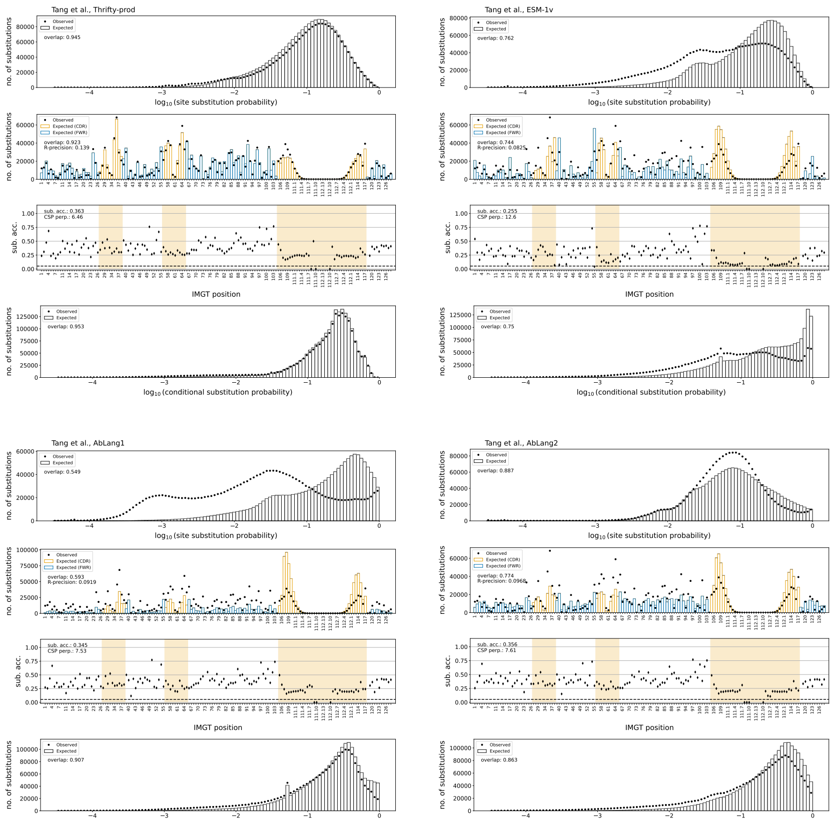

Fig B. AA framework models on the Tang et al. data set. For each model, there are four panels. The top panel shows the observed and expected number of substitutions where the sites are binned according to their predicted SSP. The second panel shows the observed and expected number of substitutions over site position. The third panel shows the per-site substitution accuracy. The bottom panel shows the observed and expected number of substitutions at sites of substitution, and the sites are binned according to their predicted CSP. The hatch-filled bands are the standard deviations on the expectations.

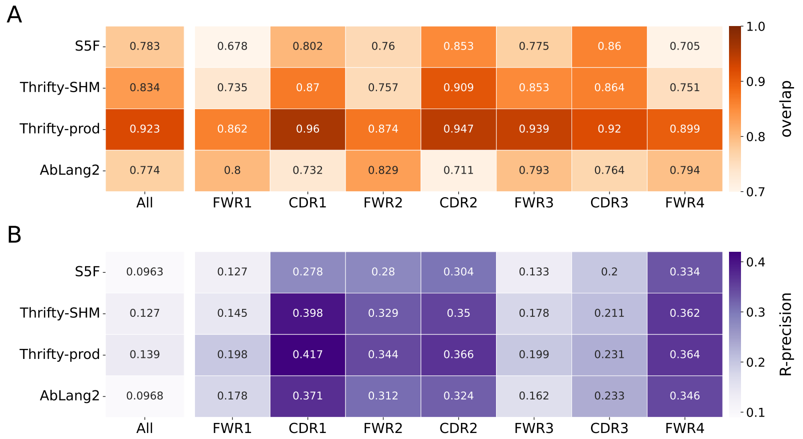

Fig C. Per-region overlap and R-precision on the Tang et al. data set. (A) Site substitution overlap. (B) R-precision.

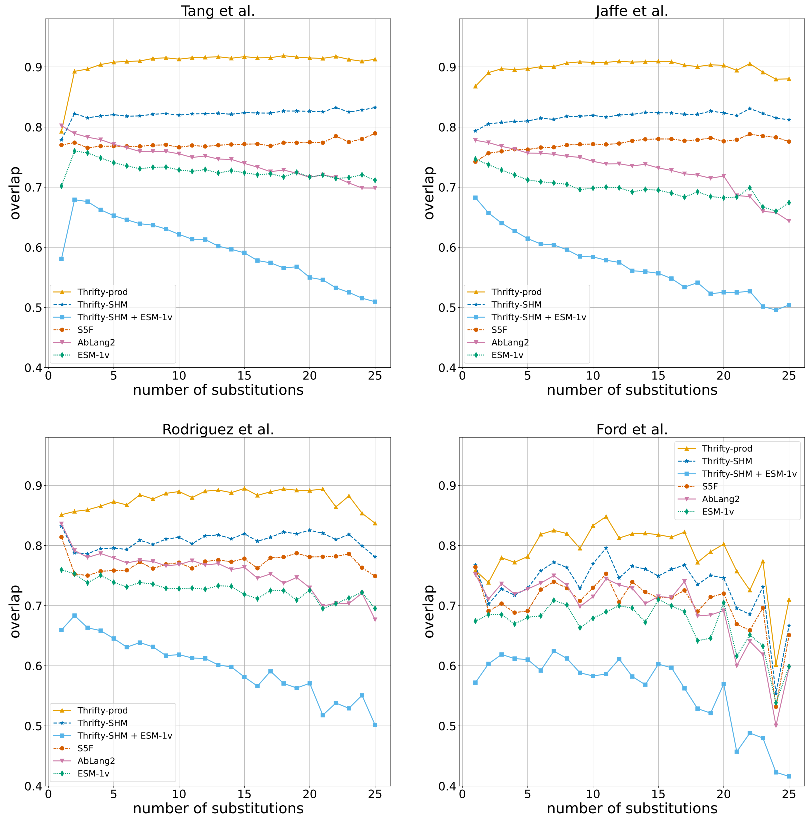

Fig D. Overlap stratified by number of substitutions per PCP evaluated on the human data sets.

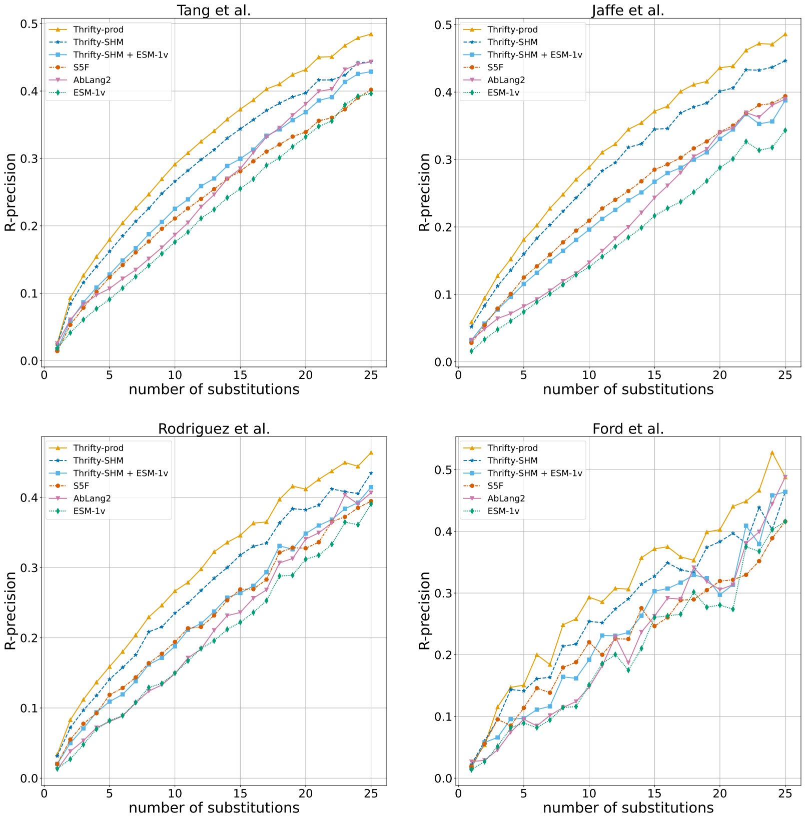

Fig E. R-precision stratified by number of substitutions per PCP evaluated on the human data sets.

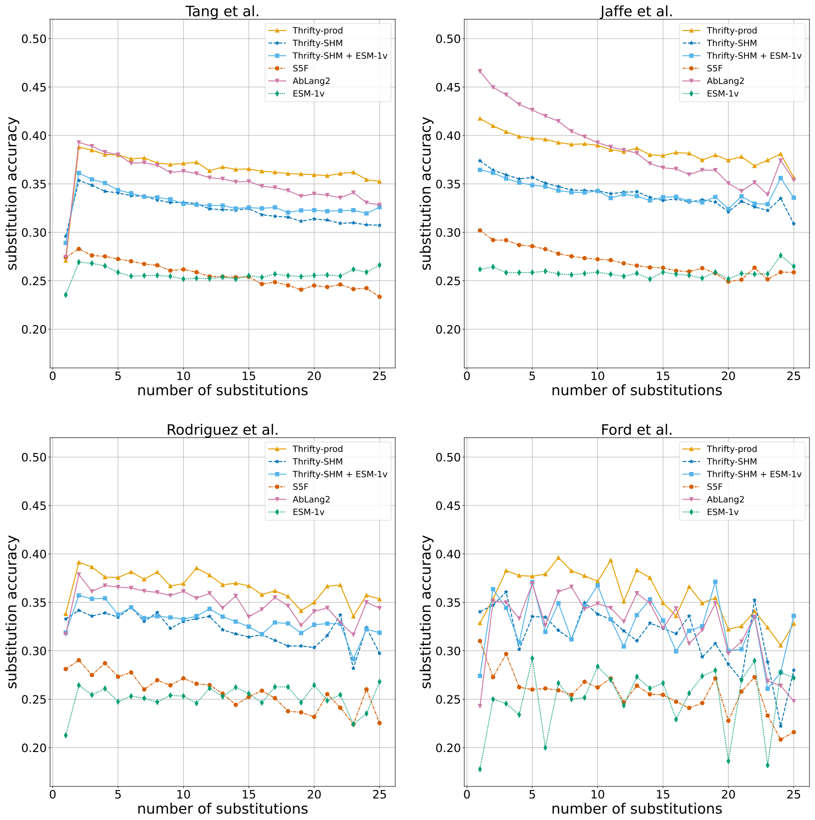

Fig F. Substitution accuracy stratified by number of substitutions per PCP evaluated on the human data sets.

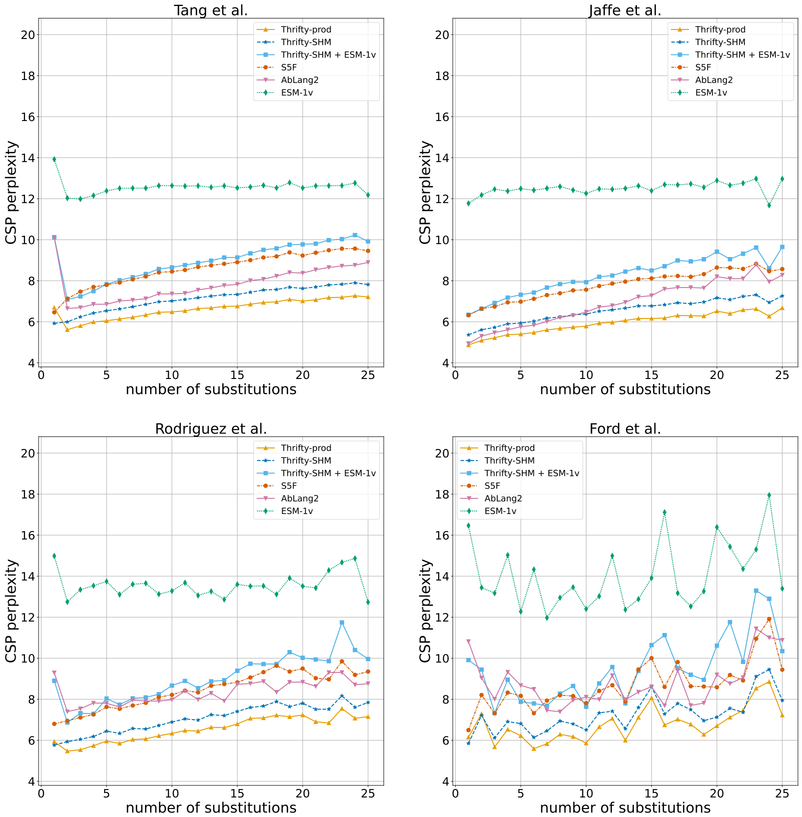

Fig G. CSP perplexity stratified by number of substitutions per PCP evaluated on the human data sets.

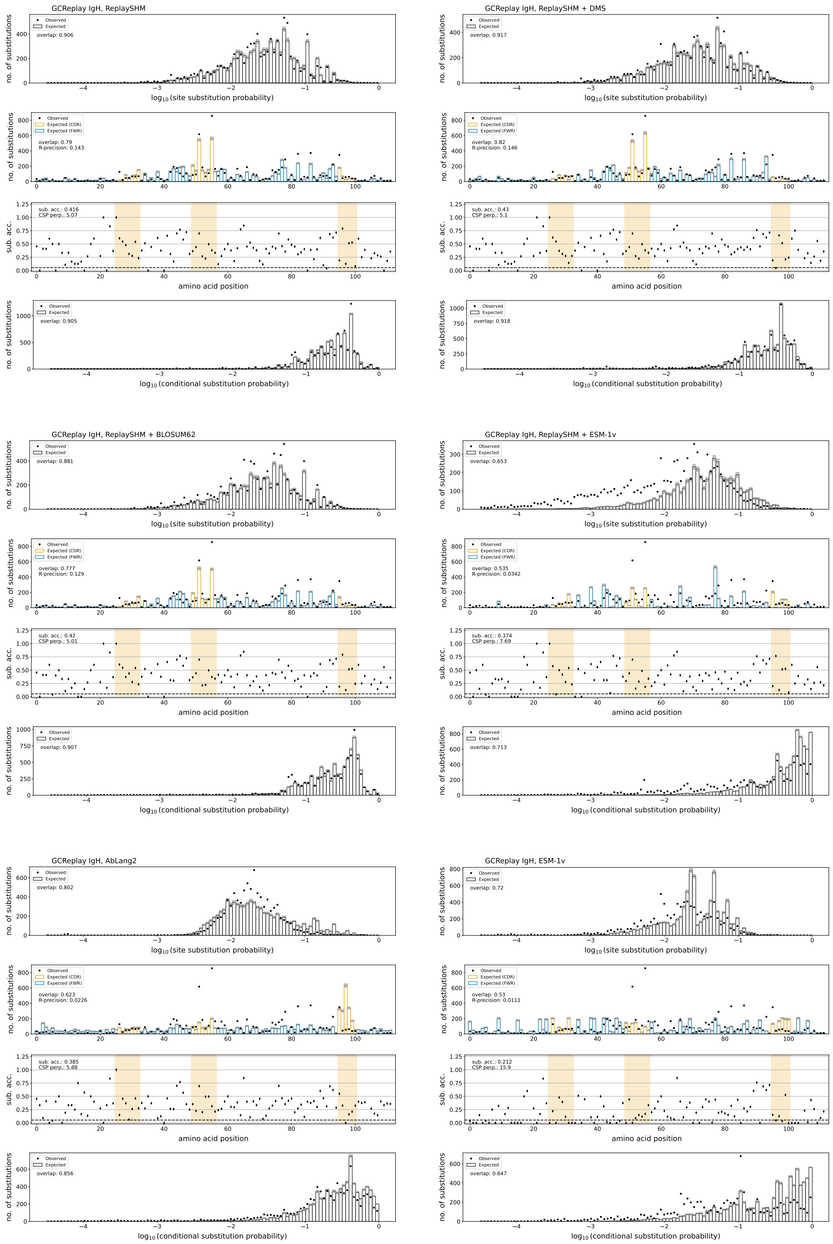

Fig H. All models on Replay heavy chain data set. For each model, there are four panels. The top panel shows the observed and expected number of substitutions where the sites are binned according to their predicted SSP. The second panel shows the observed and expected number of substitutions over site position. The third panel shows the per-site substitution accuracy. The bottom panel shows the observed and expected number of substitutions at sites of substitution, and the sites are binned according to their predicted CSP. The hatch-filled bands are the standard deviations on the expectations.

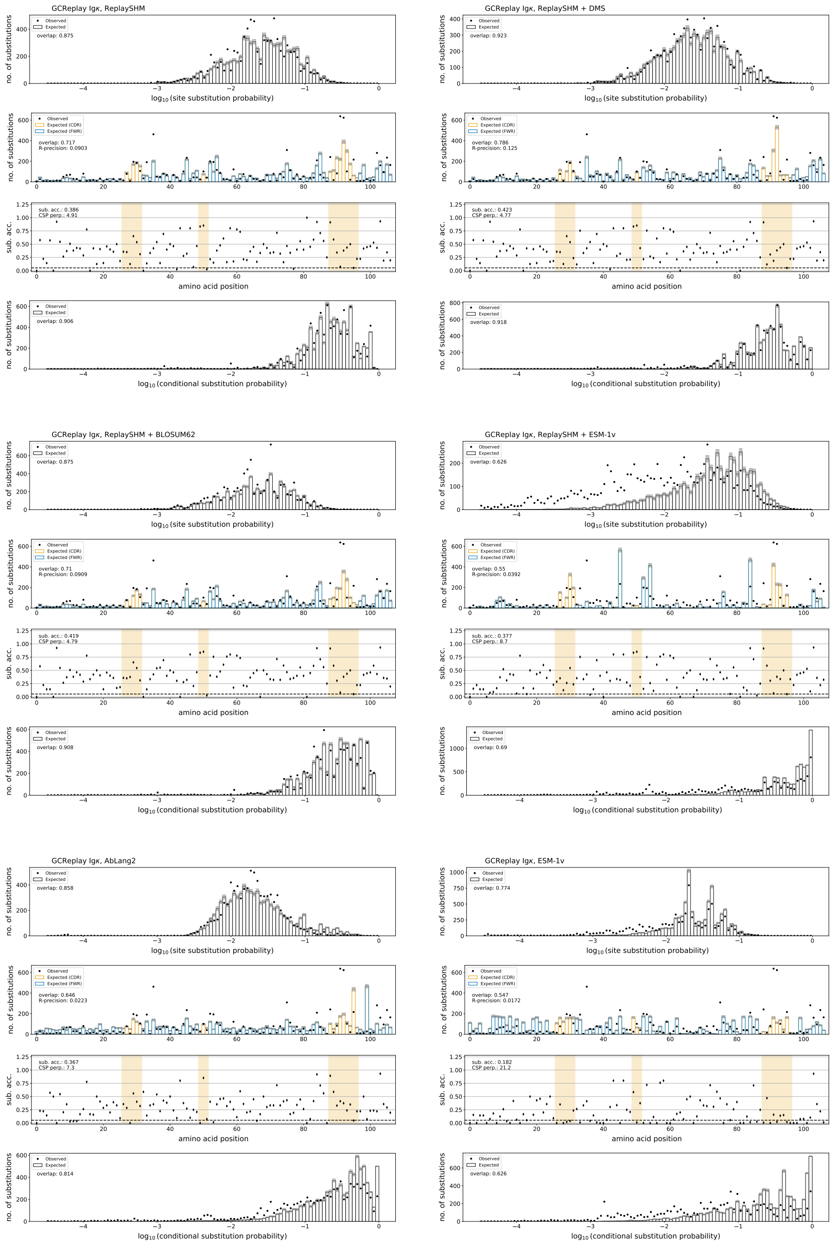

Fig I. All models on Replay light chain data set. For each model, there are four panels. The top panel shows the observed and expected number of substitutions where the sites are binned according to their predicted SSP. The second panel shows the observed and expected number of substitutions over site position. The third panel shows the per-site substitution accuracy. The bottom panel shows the observed and expected number of substitutions at sites of substitution, and the sites are binned according to their predicted CSP. The hatch-filled bands are the standard deviations on the expectations.

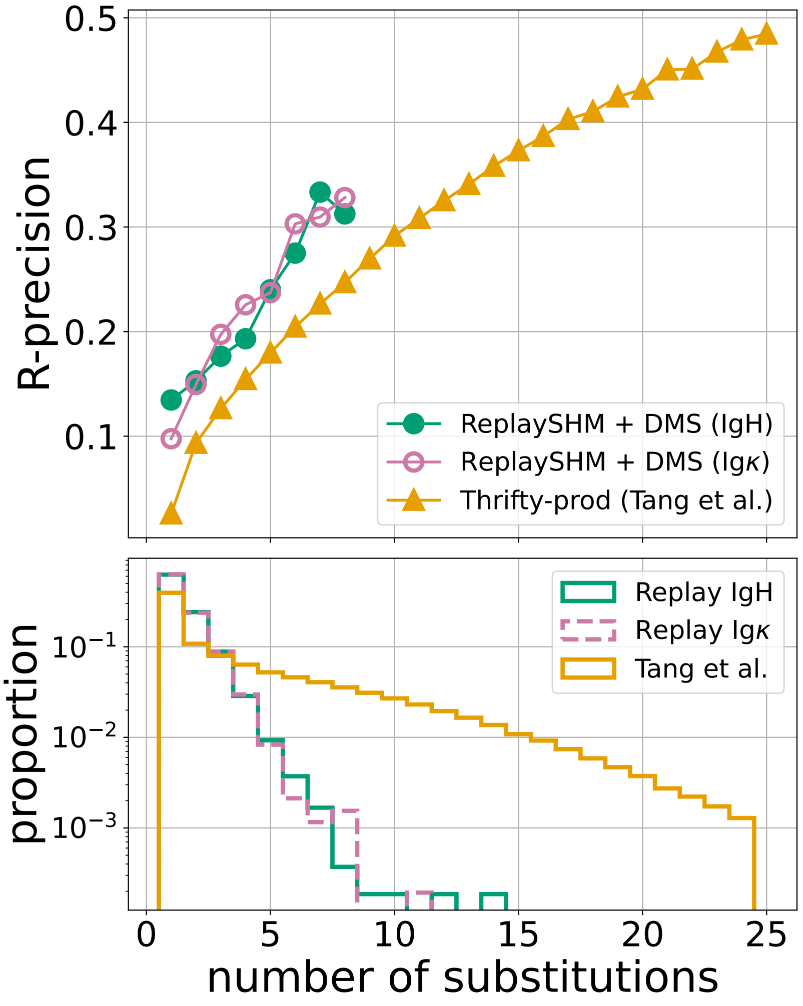

Fig J. Comparing results across human repertoires and the Replay data. (Upper): When stratified by number of substitutions per PCP, R-precision of ReplaySHM + DMS on Replay data is better than models on human repertoire data. The Thrifty-prod model is shown, which is the best model that we evaluated on human data. (Lower): Distribution of the number of substitutions per PCP in the Replay data sets and the Tang et al. data set. The Tang et al. data (and similarly in the other human repertoire data sets) consists of more PCPs with higher number of substitutions than the Replay data.

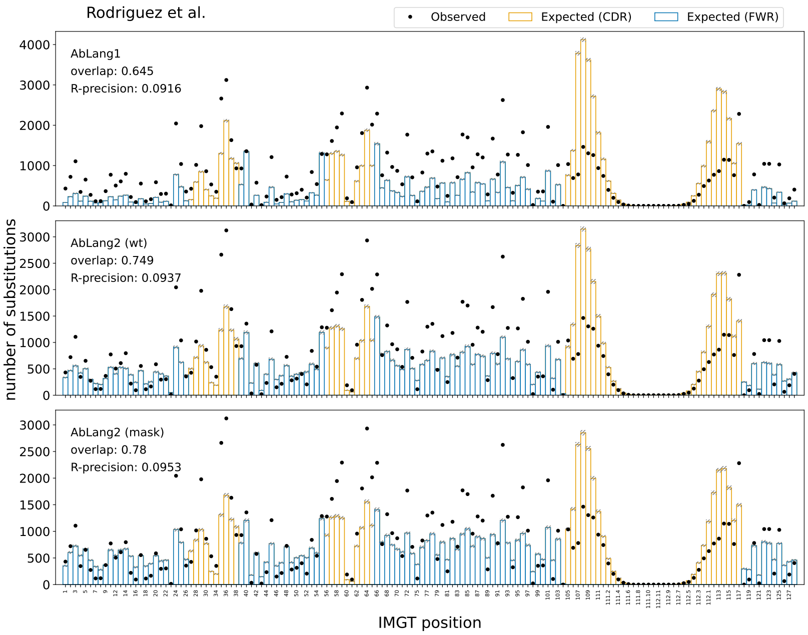

Fig K. Improved antibody prediction with AbLang2 on Rodriguez et al. IgH data set. Results are presented for AbLang1 and AbLang2 using both scoring strategies. AbLang2 (mask) achieved the best performance across all metrics, and was used in all other AbLang2 results presented.

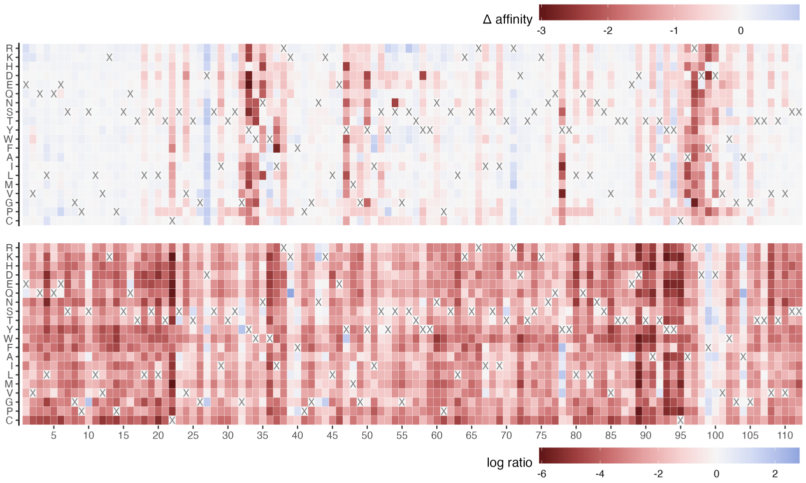

Fig L. ESM-1v predictions do not agree with DMS affinity findings in Replay IgH naive sequence. (Upper): DMS affinity measurements for the Replay IgH naive sequence. (Lower): ESM-1v predictions for the same naive sequence, presented as the log transformed probability ratio. The wildtype amino acid at each site in the naive sequence is depicted with a 'X'. Values are relative to the wildtype amino acid in the naive sequence context.

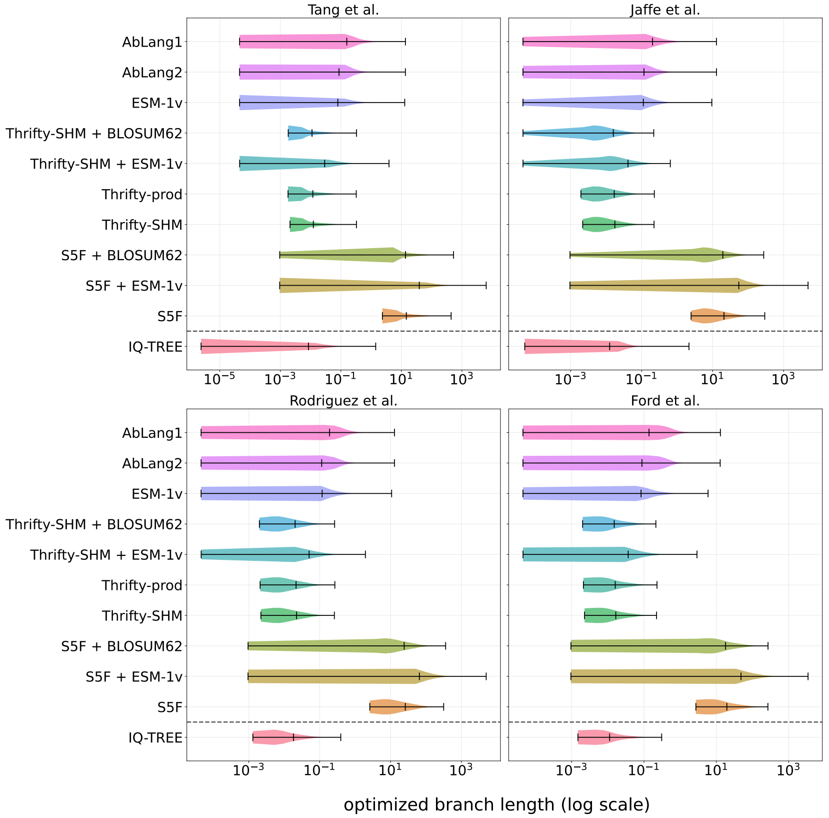

Fig M. Optimized per-PCP branch lengths ($\hat{\tau}$) vary widely across models. The distribution of $\hat{\tau}$ for PCPs under each model is shown for all human data sets. Median $\hat{\tau}$ values are indicated with vertical dashed lines. For reference, the IQ-TREE branch lengths are shown.

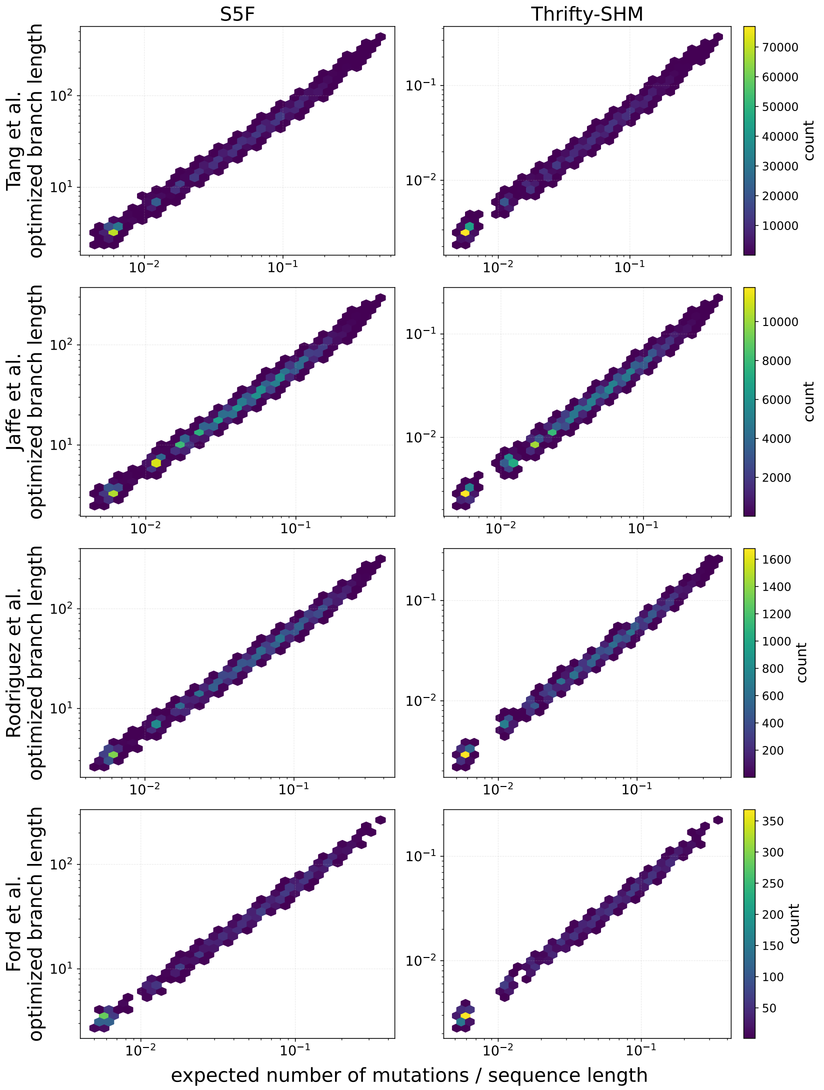

Fig N. NT framework models predict mutation to occur at frequencies below one mutation per site. For all PCPs in the human data sets, the expected number of substitutions per PCP (normalized by codon length) under the S5F and Thrifty-SHM model is plotted against the optimized branch length ($\hat{\tau}$). Colored bins indicate the density of points in the scatter plot.

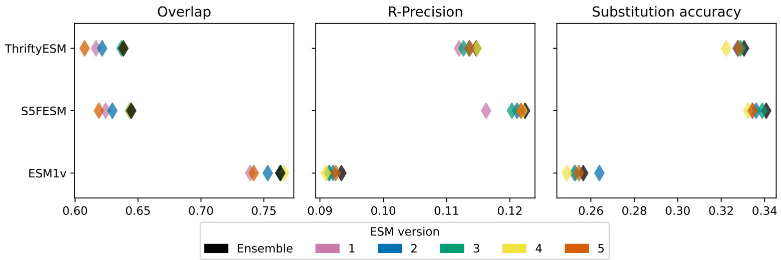

Fig O. The ensemble ESM-1v model does not offer a significant performance improvement over individual models in the Rodriguez et al. data set. For reference, the best-performing model on this data set obtained an overlap of 0.900, R-precision of 0.163, and substitution accuracy of 0.370. This trend was observed in when ESM-1v was used in a standalone model as well as when it was used to compute selection factors in the NT-AA framework. No model consistently performed the best across all metrics. Model 1 of the ESM-1v ensembles was used in all other ESM-1v results presented.

References

[1] Yaari G, Vander Heiden JA, Uduman M, Gadala-Maria D, Gupta N, Stern JNH, et al. Models of somatic hypermutation targeting and substitution based on synonymous mutations from high-throughput immunoglobulin sequencing data. Front Immunol. 2013;4:358. doi:10.3389/fimmu.2013.00358.

[2] Sung K, Johnson MM, Dumm W, Simon N, Haddox H, Fukuyama J, et al. Thrifty wide-context models of B cell receptor somatic hypermutation. eLife. 2025;14:RP105471. doi:10.7554/eLife.105471.1.

[3] Olsen TH, Moal IH, Deane CM. AbLang: an antibody language model for completing antibody sequences. Bioinform Adv. 2022;2(1):vbac046. doi:10.1093/bioadv/vbac046.

[4] Olsen TH, Moal IH, Deane CM. Addressing the antibody germline bias and its effect on language models for improved antibody design. Bioinformatics. 2024;40(11):btae618. doi:10.1093/bioinformatics/btae618.

[5] Ford EE, Tieri D, Rodriguez OL, Francoeur NJ, Soto J, Kos JT, et al. FLAIRR-seq: A method for single-molecule resolution of near full-length antibody H chain repertoires. J Immunol. 2023;210(10):1607–1619. doi:10.4049/jimmunol.2200825.

[6] Rodriguez OL, Safonova Y, Silver CA, Shields K, Gibson WS, Kos JT, et al. Genetic variation in the immunoglobulin heavy chain locus shapes the human antibody repertoire. Nature Comm. 2023;14(1):4419. doi:10.1038/s41467-023-40070-x.

[7] Jaffe DB, Shahi P, Adams BA, Chrisman AM, Finnegan PM, Raman N, et al. Functional antibodies exhibit light chain coherence. Nature. 2022;611(7935):352–357. doi:10.1038/s41586-022-05371-z.

[8] DeWitt WS, Vora AA, Araki T, Galloway JG, Alkutkar T, Bortolatto J, et al. Replaying germinal center evolution on a quantified affinity landscape. BioRxiv [Preprint]. 2025 bioRxiv 2025.06.02.656870. Available from: https://www.biorxiv.org/content/10.1101/2025.06.02.656870v1 doi:10.1101/2025.06.02.656870.

[9] Meier J, Rao R, Verkuil R, Liu J, Sercu T, Rives A. Language models enable zero-shot prediction of the effects of mutations on protein function. Adv Neural Inf Process Syst. 2021;34:29287–29303.

[10] Notin P, Kollasch A, Ritter D, van Niekerk L, Paul S, Spinner H, et al. ProteinGym: Large-Scale Benchmarks for Protein Fitness Prediction and Design. In: Oh A, Naumann T, Globerson A, Saenko K, Hardt M, Levine S, editors. Adv. Neural Inf. Process. Syst.. vol. 36; 2023. p. 64331–64379. Available from: `https://proceedings.neurips.cc/paper_files/paper/2023/`

file/cac723e5ff29f65e3fcbb0739ae91bee-Paper-Datasets_and_Benchmarks.pdf.

[11] Tang C, Bagnara D, Chiorazzi N, Scharff MD, MacCarthy T. AID overlapping and Polη hotspots are key features of evolutionary variation within the human antibody heavy chain (IGHV) genes. Front Immunol. 2020;11:788. doi:10.3389/fimmu.2020.00788.

[12] Henikoff S, Henikoff JG. Amino acid substitution matrices from protein blocks. Proceedings of the National Academy of Sciences. 1992;89(22):10915–10919. doi:10.1073/pnas.89.22.10915.
