# Supplementary material for: Nucleotide context models outperform protein language models for predicting antibody affinity maturation
Source: PLoS Comput Biol. 2025 Dec 1;21(12):e1013758. doi: 10.1371/journal.pcbi.1013758 (PMC12685221; doi:10.1371/journal.pcbi.1013758)
Supplement: Attachment — Submitted filename: rebuttal.pdf [file pcbi.1013758.s003.pdf]

Response to reviews for "Nucleotide context models outperform protein language models for predicting antibody affinity maturation"

We thank the editor and reviewers for their time spent on evaluating our manuscript and providing many helpful comments. The comments have led us to do a number of additional analyses that have strengthened the manuscript.

Below, we explain how we addressed each comment raised by the editor and reviewers. Statements from the editor and reviewers are presented in **bold** font. New or updated text in the revised manuscript are presented in *italics* font.

Additional Editor Comments

One of the critical comments to address is novelty of the proposed model; including a detailed comparison with ARMADiLLO, as proposed by one of the reviewers, makes sense.

The comparison with ARMADiLLO is provided below in our response to the reviewer.

Reviewer #1

In there manuscript "Nucleotide context models outperform protein language models for predicting antibody affinity maturation" Johnson et al. develop and benchmark a model taking nucleotide sequence context into processes for modelling of affinity maturation. This approach has scientific relevance as hypermutation is strongly influenced by the nucleotide sequence context both in terms of promoting or preventing substitution. The authors conclude that such an approach performs better than other models. The authors might however also consider the following aspects: In the past, the ARMADiLLO tool (e.g. 10.1093/nar/gkad398) uses a similar concept to assess evolution of antibodies. How do these methodologies compare in terms of accuracy of evolution prediction?

Thank you for this suggestion. The ARMADiLLO framework computes probabilities of amino acid outcomes under the SHM-only scenario, based on the S5F model. In our manuscript, we also compare amino acid outcomes under the S5F model. Both frameworks account for evolutionary time along a parent-child pair (PCP): in ARMADiLLO, the sequences are simulated based on S5F mutabilities to match the observed number of mutations, while in our framework we optimize a likelihood based on S5F mutabilities. This is a minor difference, and if anything we have a slightly more sophisticated approach, as we take mutation rates into account and use likelihoods. We have added text to 'Discussion' to acknowledge their work and similarities with EPAM:
Our approach to calculate probabilities of amino acid outcomes from nucleotide-based predictions of SHM has similarities to the ARMADiLLO framework. That approach generates predictions under the S5F model of SHM while matching the number of mutations in a PCP, while we match branch lengths. Because branch lengths take rate into account, this should result in slightly better performance for the NT framework.

Evolution does, as the authors predict, not only depend on the linear protein sequence but also on the nucleotide sequence that encodes it. However, evolution also depends on the structural context that neither methodology really approach. Some residues (e.g. but not limited to C41, W41, W52, and C104) are so important for the structure that they never evolve. Others may evolve but are restricted to evolution to conserved substitutions (e.g. L, I, M; nucleotide sequence prediction softwares (e.g. ARMADiLLO) would typically overestimate substitutions at such sites) while others are able to accommodate essentially any residue. Other parts, not specifically discussed, of the domains, such as the upper and lower cores, may have similar restrictions. In some cases, germline genes encode unusual resides (not encoded by other genes, such as residues 46 and 81 of human IGHV1-8-derived antibodies) that are highly mutated (see for instance DOI: 10.3389/fimmu.2017.01433), sites that may be missed by a nucleotide-focused prediction tools. The authors ought to discuss the structural context and its role in prediction. Are combinations of models as applied here better able to incorporate the structural perspective than without the need for explicitly incorporating a structural component into the computational process?

We agree that structural context is an important consideration for amino acid outcomes in affinity maturation. For this reason, we investigate the predictive performance of models at highly conserved sites such as C23, W41, C104, and W118 (Fig 2A and S7 Table), as well as evaluate performance split into CDR and FWR regions (S3 Fig and Fig 3). As the reviewer predicts, we find that nucleotide-based models (S5F and Thrifty-SHM) substantially overestimate the number of substitutions at conserved sites, while the protein language models performed better but still overestimated. Specifically responding to the question of whether combinations of models can better incorporate structural perspective, we find that for conserved residues, AbLang2 and ESM-1v perform better than the ME models that combine mutation and selection, with AbLang2 providing relatively accurate estimates for C23 and C104 but overestimating other sites. However, the performance of all of these models leaves much to be desired, highlighting the need for future work.

We have added comments on this issue in 'Discussion':
None of the models we evaluated explicitly incorporate structural information, although masked language models have demonstrated some ability to learn structural constraints from sequence alone. Our empirical analysis at highly conserved residues reveals that this implicit learning is insufficient: while AbLang2 and ESM-1v perform better than SHM-only models, they still substantially overestimate substitutions at structurally constrained sites such as W41, W52, and W118. Unsurprisingly, combining these models with mutation models does not successfully capture structural constraints either, performing worse than protein language models at conserved sites.

The authors removed sequences that had >10 mutations in any window of 20 consecutive sites (lines 442-445). I understand the rationale but how does this impact in particular analysis of diversification of CDRs where higher levels of diverse substitutions may be observed. Please discuss.

This is a good point! We find our overlap, substitution accuracy, and CSP perplexity metrics vary only mildly at high number of substitutions so that the removal of truly highly diversified sequences should have little impact on our analysis. We added text to discuss this in 'Materials and methods':
While this may remove sequences that are truly highly diversified, for instance in the CDRs, it should have little impact on our analysis because the overlap, substitution accuracy, and CSP perplexity metrics showed mild variation at high number of substitutions (S4, S6, S7 Figs).

Minor comments

Lines 338-346 appears to be Discussion and should be moved to that section.

We have moved this to 'Discussion', thanks.

The authors should indicate the reference sets (incl version number) that was used to annotate germline gene origin.

The human germline reference sets are from OGRDB: IGH_VDJ ver 7 (10.5281/zenodo.8305006), IGKappa_VJ ver 2 (10.5281/zenodo.8305008), and IGLambda_VJ ver 1 (10.5281/zenodo.8305013). We now state these in 'Materials and methods' and added the corresponding citations:
Germline gene annotations are based on OGRDB human germline sets IGH_VDJ ver 7, IGKappa_VJ ver 2, and IGLambda_VJ ver 1.

Have models been trained solely on sequences derived from the same germline gene or also on sequences derived from other genes? Residues far apart in the linear sequence may come into close proximity in the folded protein and this may impact mutability in particular of residues that are structurally important. Please comment.

Models are trained on repertoire data and therefore span the collection of genes present in individuals. With respect to protein folding, we agree and have addressed this comment with manuscript edits above.

Reviwer #2

Timely benchmark: EPAM (code released) shows nucleotide-context SHM models outperform protein LMs across human repertoires and a Replay system. With the revisions, this would be an important community reference.

This manuscript introduces EPAM, a unified benchmarking framework for evaluating models that predict antibody affinity maturation along BCR lineage branches. The central result is that nucleotide-context SHM models, especially a wide-context CNN, consistently outperform state-of-the-art protein language models (including an antibody-specific LM) at predicting (i) which sites mutate and (ii) the resulting amino-acid outcomes. The evaluation spans large human repertoire datasets and a controlled, antigen-specific mouse Replay system; code is released and data are to be slated for Zenodo.

It's timely, careful work that nudges the field away from amino-acid-only analysis and toward an obvious-but-overlooked point that nucleotide context matters for BCR evolution. A few design choices could bias the comparisons, though, and several robustness/calibration checks would strengthen the generality of the claims.

We thank you for your positive comments.

Major comments

1. EPAM fits an effective evolutionary time ($\hat{\tau}$) per parent-child pair to maximize model likelihood, then evaluates at that $\hat{\tau}$. This puts all models in their best light, but it may also let misspecified models compensate by stretching/shrinking τ. Please add controls: (i) fix τ to the phylogeny's branch length, (ii) fix τ per lineage, and (iii) stratify results by inferred branch-length bins. Showing that conclusions hold without per-PCP $\hat{\tau}$ would remove a key concern.

Thank you for your suggestions. Indeed, the approach allows misspecified models to achieve better performance. However, not doing so would result in apparent differences that are more incidental rather than integral to the models.

Indeed, in our examinations of optimized values $\hat{\tau}$ relative to each other and the branch length from IQ-TREE, we see that S5F models vs Thrifty vs MLMs have distinct differences in their distributions of branch lengths, varying by orders of magnitude between S5F and MLMs. Thrifty most resembles the IQ-TREE branch lengths, although this is to be expected because it was developed in this evolutionary framework. This also implies that control scenario (i) would most negatively impact the performance of S5F and MLM models. For control scenario (ii), it is challenging to choose a fixed value of τ

per lineage that would allow fair comparisons between models. Regarding (iii), we have computed model performance stratified by number of amino acid substitutions (S4–S7 Figs), and found the relative performances between models remain consistent. Since branch length is highly correlated with number of substitutions, these figures should be a good proxy for control scenario (iii).

We have added a supplemental figure showing optimized branch lengths for each model relative to each other and IQ-TREE as well as the following text to the 'Materials and methods' section:
This value, $\hat{\tau}$, varied widely across models for a given PCP (S13 Fig). Optimized values for Thrifty-SHM and Thrifty-prod were closest to the branch length inferred IQ-TREE. This design choice allowed all models to be evaluated in their best light.

2. The NT framework converts context-dependent nucleotide rates into codon/AA probabilities while assuming at most one mutation per site along a PCP. The paper argues this is reasonable because branches are short; please quantify: show the distribution of per-site expected hits under your λ estimates and re-run metrics after removing the longest x% of branches. If feasible, add a CTMC sanity check for a subset.

We have taken a closer look at this assumption in our nucleotide-context models (S5F and Thrifty-SHM). In comparing the expected number of mutations for these models relative to the given PCP's sequence length, we find that these models predict mutation rates well below this assumption. We have added the following statement to 'Methods and materials' as well as the referenced supplemental figure:
We confirmed that the expected number of mutations for PCPs under NT models was far below one mutation per site for all human data sets considered (S14 Fig).

Also, given that branch length is strongly correlated with expected number of mutations (as seen in S14 Fig), we can see the impact of removing the highest branch lengths from those metrics in our supplemental analysis of metric breakdown by number of substitutions (S4–S7 Figs, dark blue and orange lines). While R-precision is closely correlated with number of mutations, all other metrics show only mild dependence at high number of substitutions.

3. Some human datasets overlap with OAS, which was used to train AbLang models; Thrifty-prod is trained on Jaffe et al. productive sequences. Please (a) isolate truly out-of-sample evaluations (e.g., Rodriguez, Ford) and (b) report per-dataset performance with clear "in-OAS vs out-of-OAS" flags to eliminate concerns that either model family benefits from dataset overlap. Also confirm that no sequences used to train Thrifty-prod appear in its evaluation sets.

Thank you for this important comment. We now address these points in "Overview of data preparation and EPAM model evaluation" subsection of 'Results'. After introducing the human repertoire data sets, we describe the overlap situation of Tang et al. and Jaffe et al., and also mention that the results of Tang et al. are consistent with Rodriguez et al. and Ford et al., which indicates minor impact from any biases:
It should be noted that Jaffe et al. and part of Tang et al. are in the training data set for the AbLang2 model, while Jaffe et al. is the training data set for the Thrifty-prod model (see Overview of models). We found consistent results with Tang et al., Rodriguez et al., and Ford et al.—the latter two data sets represent truly out-of-sample evaluations, suggesting that biases due Tang et al. sequences shared by training and evaluation had limited effect.

At the end of that subsection, we now reiterate that while Tang et al. is not completely independent of training data, the consistency of results across data sets supports that our conclusions based on Tang et al. are robust:
We present results in the main text for the largest human data set, Tang et al., and the Replay experiment. As stated above, the Tang et al. data set is partially included in the training data for the AbLang2 model. While Rodriguez et al. and Ford et al. do not overlap with any training data set, they have considerably fewer sequences. The results with the Rodriguez et al. (S2 Table) and Ford et al. (S3 Table) data sets were qualitatively consistent with Tang et al. (S4 Table) and indicates that

biases from overlap of training and evaluation data had little impact. In particular, the performance of AbLang2 on Tang et al. was not strikingly different from its performance on Rodriguez et al. and Ford et al.. This gives confidence that our conclusions based on Tang et al. are robust. In contrast, Jaffe et al. makes up a larger portion of the training data for AbLang2 and Thrifty-prod, and we found much better substitution accuracy and CSP perplexity for those two models on that data set (S5 Table).

We have updated S1 Table to explicitly indicate the data sets that are not in OAS nor in any training data set. The captions for the tables of human repertoire results (S2,S3,S4,S6) are updated to state if the data set overlaps with any model training data or is truly out-of-sample.

4. In the antigen-specific Replay setting, combining ReplaySHM + DMS improves location prediction and yields top substitution accuracy/perplexity, but the gains over ReplaySHM alone are modest. Please expand on why, e.g., is binding-only DMS insufficient, do epistasis or expression constraints limit single-mutant DMS transfer; are per-site SHM biases already capturing much of the signal? A quantitative decomposition (how much lift comes from which regions/sites) would help.

Thank you for asking if binding-only DMS is insufficient, which led us to do an additional analysis combining binding and expression data as described in the new 'Results' text below, which also addresses the other questions.

For the Replay data set, the ReplaySHM + DMS model performed the best, as expected. However, the gains from incorporating binding measurements to ReplaySHM were modest. We attempted to incorporate expression measurements to calculate selection factors and found the same results as with binding alone (S10 Table). Splitting the data into PCPs where the naive BCR sequence is the parent and PCPs where the child sequence is a leaf node provided some insight (S11 Table). Performance gains for ReplaySHM + DMS over ReplaySHM alone is substantially greater in the naive-parent PCPs than in the leaf-child PCPs for substitution accuracy and CSP perplexity in both heavy and light chain data, and for R-precision in light chain data. Note that less than 10% of all PCPs are naive-parent PCPs but over 70% are leaf-child PCPs. The DMS measurements were relative to the naive BCR sequence and therefore should be more accurate when applied to naive-parent PCPs than for leaf-child PCPs. Furthermore, leaf node B cells may not have been fully subjected to the selection process, consequently selection factors would play a lesser role than SHM modeling for leaf-child PCPs. Indeed, we observe that ReplaySHM performs much better on leaf-child PCPs than on naive-parent PCPs in almost all cases (the exception being R-precision for heavy chain data). Interestingly, the overlap and R-precision for heavy chain data show no gains for naive-parent PCPs and only small gains for leaf-child PCPs, suggesting the possibility that ReplaySHM already captures much of the predictive power for which sites will undergo substitution.

5. Results suggest nucleotide context governs where mutations land, while productive-sequence models (Thrifty-prod) help more with which amino acid appears. Consider adding per-region (FWR/CDR) attribution or SHAP-style analyses to support these mechanistic interpretations (e.g., motif-driven SHM vs selection constraints). This could also discuss why AbLang2 excels in Replay CDR3 accuracy yet lags elsewhere.

We added evaluation of per-region overlap and R-precision (S3 Fig). We describe these in 'Results':
Computing overlap restricted to each FWR and CDR, the SHM-only models performed much better in CDRs than in FWRs, while the trend is reversed for AbLang2 (S3 Fig). We attributed this to SHM and nucleotide context being more important in CDRs, while purifying selection and amino acid preferences are more important in FWRs. Overlaps for Thrifty-prod were relatively consistent across regions. For regional R-precision scores, AbLang2 performed similar to or better than Thrifty-SHM or S5F respectively, but over full sequences it was worse than Thrifty-SHM and equal to S5F (S3 Fig). This highlights the value of nucleotide context to predicting sites of amino acid substitutions.

Minor comments

Figure fonts are blurry and hard to read. Consider improving the resolution of figures, and increase font sizes where appropriate

The figures have been regenerated to improve resolution, thanks.

The LM citation list includes a placeholder "[?, 20–25,27]" please fix

Thank you for catching that! This has been fixed.

Define tie-breaking for R-precision when multiple sites share the same SSP.

In the event of ties for the kth highest SSP, the site is randomly selected. We have added this description:
In cases where the kth highest SSP can be multiple sites, the site is randomly selected.

Clarify whether CSP perplexity is computed over all substitution events equally or weighted by PCP; a short formula example in the main text would help.

We have added a sentence to clarify:
All substitution events are counted equally, regardless of the PCP in which they occur.

Add a short Methods paragraph on the IMGT region definitions used for FWR/CDR partitioning (numbers are implied, but a concrete table helps readers).

We added Table 1 in the 'Data preparation' subsection listing the IMGT positions for each FWR and CDR region.

Report parameter counts (already stated qualitatively) and, if possible, compute cost for each model to underscore the "small CNN beats huge LM" message.

The number of model parameters are reported in the "NT framework" subsection of 'Results':
There are 5,931 parameters in Thrifty-SHM, which is about twice the number of parameters in S5F (3,072), but orders of magnitude less than protein sequence language models like AbLang2 (45 million) and ESM-1v (650 million).

Consider moving one or two Replay performance tables/plots from the supplement into the main text.

We have moved the heavy chain Replay performance plot from supplement to main body.
