# Supplementary material for: Nucleotide context models outperform protein language models for predicting antibody affinity maturation
Source: PLoS Comput Biol. 2025 Dec 1;21(12):e1013758. doi: 10.1371/journal.pcbi.1013758 (PMC12685221; doi:10.1371/journal.pcbi.1013758)
Supplement: Attachment — Submitted filename: Final_version_comments_20Aug2025.docx [file pcbi.1013758.s002.docx]

This manuscript introduces EPAM, a unified benchmarking framework for evaluating models that predict antibody affinity maturation along BCR lineage branches. The central result is that nucleotide-context SHM models, especially a wide-context CNN, consistently outperform state-of-the-art protein language models (including an antibody-specific LM) at predicting (i) which sites mutate and (ii) the resulting amino-acid outcomes. The evaluation spans large human repertoire datasets and a controlled, antigen-specific mouse Replay system; code is released and data are to be slated for Zenodo.

It’s timely, careful work that nudges the field away from amino-acid–only analysis and toward an obvious-but-overlooked point that nucleotide context matters for BCR evolution. A few design choices could bias the comparisons, though, and several robustness/calibration checks would strengthen the generality of the claims.

**Major comments:**

1. EPAM fits an effective evolutionary time (τ̂) per parent–child pair to maximize model likelihood, then evaluates at that τ̂. This puts all models in their best light, but it may also let misspecified models compensate by stretching/shrinking τ. Please add controls: (i) fix τ to the phylogeny’s branch length, (ii) fix τ per lineage, and (iii) stratify results by inferred branch-length bins. Showing that conclusions hold without per-PCP τ̂ would remove a key concern.
2. The NT framework converts context-dependent nucleotide rates into codon/AA probabilities while assuming at most one mutation per site along a PCP. The paper argues this is reasonable because branches are short; please quantify: show the distribution of per-site expected hits under your λ estimates and re-run metrics after removing the longest x% of branches. If feasible, add a CTMC sanity check for a subset.
3. Some human datasets overlap with OAS, which was used to train AbLang models; Thrifty-prod is trained on Jaffe et al. productive sequences. Please (a) isolate truly out-of-sample evaluations (e.g., Rodriguez, Ford) and (b) report per-dataset performance with clear “in-OAS vs out-of-OAS” flags to eliminate concerns that either model family benefits from dataset overlap. Also confirm that no sequences used to train Thrifty-prod appear in its evaluation sets.
4. In the antigen-specific Replay setting, combining ReplaySHM + DMS improves location prediction and yields top substitution accuracy/perplexity, but the gains over ReplaySHM alone are modest. Please expand on why, e.g., is binding-only DMS insufficient, do epistasis or expression constraints limit single-mutant DMS transfer; are per-site SHM biases already capturing much of the signal? A quantitative decomposition (how much lift comes from which regions/sites) would help.
5. Results suggest nucleotide context governs *where* mutations land, while productive-sequence models (Thrifty-prod) help more with *which* amino acid appears. Consider adding per-region (FWR/CDR) attribution or SHAP-style analyses to support these mechanistic interpretations (e.g., motif-driven SHM vs selection constraints). This could also discuss why AbLang2 excels in Replay CDR3 accuracy yet lags elsewhere.

**Minor comments**

•Figure fonts are blurry and hard to read. Consider improving the resolution of figures, and increase font sizes where appropriate

• The LM citation list includes a placeholder (“[?, 20–25,27]”); please fix.

• Define tie-breaking for R-precision when multiple sites share the same SSP.

• Clarify whether CSP perplexity is computed over all substitution events equally or weighted by PCP; a short formula example in the main text would help.

• Add a short Methods paragraph on the IMGT region definitions used for FWR/CDR partitioning (numbers are implied, but a concrete table helps readers).

• Report parameter counts (already stated qualitatively) and, if possible, compute cost for each model to underscore the “small CNN beats huge LM” message.

• Consider moving one or two Replay performance tables/plots from the supplement into the main text.
